# Magnetic Resonance Spectroscopy – a non-invasive method in evaluating focal and diffuse central nervous system disease

**Published:** 2012-12-25

**Authors:** C Scheau, EM Preda, GA Popa, AE Ghergus, RA Capsa, IG Lupescu

**Affiliations:** Department of Radiology and Medical Imaging, “Fundeni” Clinical Institute, “Carol Davila” University of Medicine and Pharmacy, Bucharest, Romania

**Keywords:** Magnetic Resonance, MR Spectroscopy, neuroimaging, metabolic diseases, cerebral masses

## Abstract

Magnetic Resonance Spectroscopy is a non-invasive method, which can be performed following a routine Magnetic Resonance investigation within the same examination, and can provide very useful molecular information related to the metabolism and function of the normal and pathological structures of the brain. Its role is increasing in the establishment of a clear diagnosis, in both focal and diffuse central nervous system diseases, and the tendency is to replace the histopathology test, in certain cases, with similar or sometimes better diagnostic accuracy. This paper summarizes the principle, method, and main clinical applications, standing as a guide to procedure performing and results interpretation.

## Introduction

Magnetic Resonance Imaging (MRI) is the investigation of choice in the evaluation of the central nervous system, due to its invaluable morphological and functional details provided. In the last years, the development of technology and applications for this imaging modality made it possible to assess different parameters such as blood flow, water diffusion, tissue perfusion, as well as various mineral deposits [**1**]. In addition, Magnetic Resonance Spectroscopy (MRS) provides qualitative and quantitative information about molecular structures, thus being regarded as a method of molecular imaging. MRS produces a spectre of resonances correspondent to a series of metabolites, in a system of two axes, which represent the intensity of the signal (vertical axis) and the position of the signal in the frequency scale (horizontal axis, respectively), expressed in parts per million (ppm) [**2**]. The spectrum is measured within a volume of interest (VOI), which is defined on the morphological multiplanar sequences previously acquired during the examination.

## MR Spectrum

**Fig. 1:**
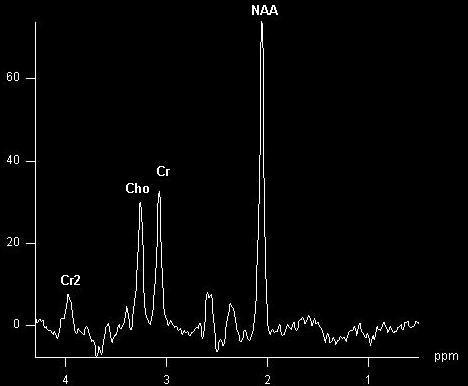
Intermediate TE acquisition (TE=135ms) in a normal patient showing prominent NAA, Cr and Cho peaks. Also, a second Cr peak at ~ 4 ppm.

Even with the new advances in technology, including the development of higher magnetic fields (such as 3T, 7T and even 9,4T), the number of molecules which can be delineated on cerebral MRS is limited [**3**]. Moreover, the quality of the spectrum is more dependent on the field homogeneity than its intensity, although high field MRS provides a better signal separation [**4**]. The metabolites present in the spectrum also depend on the echo-time (TE) of the MRS acquisition, as it follows:

 ∘ Intermediate to long TE acquisitions (144-288 ms) imply the detection of a rather poor number of metabolites (mainly N-Acetyl Aspartate (NAA), creatine (Cr) and choline (Cho), and also lactate (Lac) in pathological conditions), but with a “cleaner” spectrum, without “noise” correspondent to other molecules (**Fig. 1**).

**Fig. 2:**
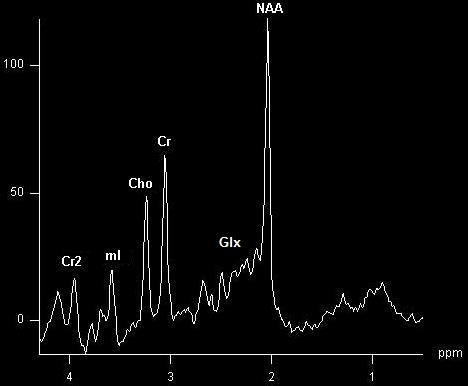
Short TE acquisition (TE=30ms) in a normal patient showing the same NAA, Cr, Cr2 and Cho peaks, and additional mI and Glx peaks, in normal ranges.

 ∘ Short TE acquisitions (<40 ms) display a wider spectrum, in addition to the previously named molecules, one can also find myo-inositol (mI), the complex formed by glutamine, glutamate and GABA (Glx), as well as free lipids, in pathological conditions. The downfall is that certain resonances overlap, posing an inconvenient in the interpretation. This can be partly resolved in high field acquisitions that increase the resolution if the field homogeneity is respected [**5**] (**Fig. 2**).

## Metabolites in normal MRS and their significance

*NAA* (2,02 ppm): one of the main amino acids in the central nervous system, and the largest signal of the spectrum, and is considered to be a marker of neuronal viability, due to the fact that it is found almost exclusively in neurons. On the other hand, some studies report that NAA may be found in oligodendrocytes or mast cells, although in minute concentrations, with a yet undetermined role [**6**]. Nevertheless, it mainly corresponds to normal cellular functionality and is diminished in tumoral or necrotic processes, multiple sclerosis, AIDS or temporal lobe epilepsy, or more generally speaking, in neural damage or replacement. In some of the conditions mentioned above, the changes may be reversible [**7,24**].

*Cr* (3,03 ppm): total Creatine is a peak which sums up creatine and phosphocreatine, very similar compounds which cannot be distinguished even in high field machines. Creatine is considered a marker of intact brain energy metabolism, as it is involved in the generation of ATP. It is also used as a reference molecule, as it is considered stable, and its concentration in different areas of the brain is well documented. Creatine is not produced in the brain, so a wide range of liver or renal diseases can lead to a decrease in the Cr peak [**8**]. As for local causes, Creatine may be reduced in tumors with increased metabolic activity like high-grade gliomas [**9**].

*Cho *(3,22 ppm): total Choline is a marker for cell wall integrity, as choline is a precursor for cellular membranes that is not embedded in the macromolecules on the membrane surface. Thus, it remains free and can be identified at MRS [**10**]. The signal increases in cases of cellular proliferation, such as malignant tumors, as well as in demyelination or inflammation or various other causes of cellular wall destruction (axonal injury) [**11,24**]. A high dietary intake of choline usually correlates with an increased correspondent peak at MRS.

*Glx *(2,05-2,50 ppm): is a composite peak, which incorporates Glutamate, Glutamine and Gamma-aminobutiric acid (GABA). These three compounds are regarded as a complex because they are not separately identifiable at 1.5T, higher fields of 4T or above being able to show some separation [**12**]. Glutamate is the main excitatory neurotransmitter in the central nervous system, and also the most abundant amino-acid in the diet. It is also involved in memory and learning, but can produce excitotoxicity in very high concentrations [**13**]. Glutamate and glutamine are in a dynamic balance, in a series of energy consuming complex reactions that occur in the neurons and in the astrocytes, respectively: glutamine is converted to glutamate in the neurons, released then uptaken by the astrocytes, which convert it back to glutamine. In opposition, GABA is the main inhibitory neurotransmitter, being involved in the pathogenesis of a series of neurological disorders such as epilepsy, schizophrenia and many others [**14,15**].

*mI *(3,56 ppm): myo-inositol is considered an astrocyte marker. mI is a pentose sugar synthesized by the glial cells, and increased values of mI correlate with an increased population of glial cells, which may occur in inflammation. Its values decrease in hepatic encephalopathy, as it is consumed in the compensation of the toxic metabolites, which have crossed the blood-brain barrier [**16**].

*Lipids* (0,8-1,5 ppm): can be present in normal conditions, when the voxel is very close to the scalp, and some fatty tissue is included in the region of interest. The lipids peak may be increased in pathological conditions such as cellular necrosis (as occurs in high-grade tumors or ischemia).

*Residual water* (4.7 ppm).

**Second peaks** of some of the above substances may be found at the following positions: *NAA *(2,6ppm), *Glx* (3,65-3,8ppm), *Cr* (3.9 ppm),* mI* (4.06 ppm).

Others assignments [**15**]:

*Propylene glycol* (1,14 ppm): may be present as a part of vehicle for drugs like phenobarbiturate.

*Ethanol *(1,16 ppm)= ethylic alcohol.

*Acetate* (1,9 ppm): can be observed in abscesses.

*Lac *(1,33 ppm): lactate is normally detected only in minimal concentrations, its rise being associated with anaerobic glycolysis (in conditions such as hypoxia or ischemia), or with areas of poor wash-out like cysts or tumors with cystic or necrotic composition [**17**]. The peak’s projection varies at different TE, in short or long TE it appears as a positive double peak, when at intermediate TE it projects as a double peak but on the other side of the baseline.

*Alanine *(1,48 ppm): can also be found in abscesses, but also in meningioma, or dying tissue.

*Acetone* (2,22 ppm): high peaks in ketogenic diet or ketoacidosis.

*Aceto-acetate *(2,29 ppm): high peaks in ketogenic diet or ketoacidosis.

*Succinate* (2,4 ppm): identifiable in abscesses, or in inborn errors of metabolism.

*Methylsulfonylmethane* (3,15 ppm): or MSM is a dietary supplement.

*Scyllo-inositol *(3,36 ppm)-sI: is an isomer of myo-inositol.

*Taurine* (3,4 ppm): can be characteristically isolated in medulloblastoma, which is of great help in the differential diagnosis from cerebellar astrocytoma [**18**].

*Glucose* (3,43 and 3,8 ppm): increased values found in diabetes mellitus, correlated with the pathognomonic hyperglycemia.

*Mannitol* (3,78 ppm): drug that acts as a vasodilator, used mainly to reduce pressure in the cranium.

*Lactate quartet* (4,11 ppm): it is an end product of anaerobe metabolism; it can be found specifically in mitochondrial damage, absence of pyruvate dehydrogenase or acceleration of glycolysis in tumors.

** Normal MRS** varies according to the following: age (0-2 years, child, adult, elderly adult) and with the position of the VOI (different MRS in gray and white matter.

## Main clinical applications

The major applications of MRS are by focal lesions (abscess vs. cystic/necrotic tumors, toxoplasmosis vs. lymphoma in HIV+ patients, unexplained lesions), metabolic disease (Creatine deficiency syndromes), hypoxia (neonatal hypoxia), trauma (prognosis in comatose patients), and dementia (Alzheimer’s disease vs. other dementia).

**Fig. 3:**
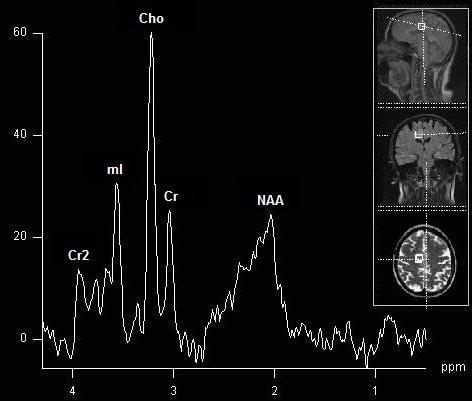
Short TE spectrum of a low grade tumor (glioma). Note the elevation of Cho and decrease of NAA. No lipids or Lac increase (absence of necrosis). Voxel placement in three-dimensional planes (T2*, Flair and T2 respectively).

*Focal anomalies.* Cerebral masses may be assessed through MRS, as it can provide information useful in the positive and differential diagnosis. It is intuitive that the voxel is placed on the lesion, but difficulties may occur when there is an inhomogeneity of structure, for necrotic and cystic areas, hemorrhage, calcifications, as well as edema should be avoided. In most cases, the voxel should encompass as much of the tissular mass as possible, in the most representative area, to get the most relevant spectrum and, in effect, the maximum diagnostic information. Acquisitions can also be performed after intravenous contrast administration, allowing a better differentiation of the tumoral area from the adjacent structures. Typical MRS anomalies in brain tumors include a decrease in NAA, and an increase in Cho (due to cellular proliferation), and lipids and lactate (in necrotic regions) (**Fig. 3**). Furthermore, a series of cerebral masses have distinct features, such as a mI increase in low-grade glioma [**19**], alanine increase in meningioma [**20**], or free amino acids in pyogenic abscesses [**21**].

**Fig. 4:**
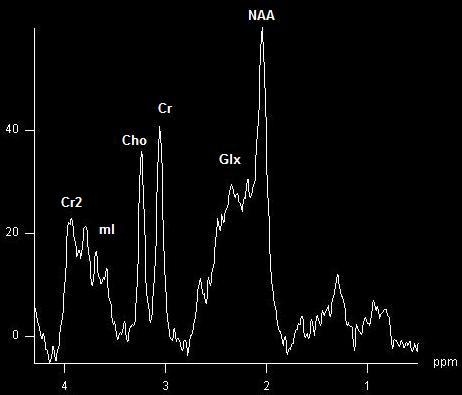
Short TE spectrum with specific changes of hepatic encephalopathy = elevated Glx and decreased mI peaks. Patient showed mild to moderate clinical symptoms.

*Diffuse anomalies.* In this case, depending on the pathology studied, we should orient our region of interest depending on the areas which are predominantly involved in that particular pathology, for instance: the posterior cingulate gyri and inferior precunci for Alzheimer Disease [**22**], hippocampus for epilepsy [**23**], parietal white matter for hepatic encephalopathy [**24**] (**Fig. 4**)), or parietal grey and white matter for dementia [**25**], with specific metabolites variations.

## Conclusion

A series of central nervous system pathological entities have specific, pathognomonic MRS changes in metabolites. MRS is a useful investigation, which can conveniently be performed following a routine MR examination, and can provide very relevant information and help lead to a concrete diagnosis.

**Acknowledgement:** This paper is supported by the Sectoral Operational Programme Human Resources Development (SOP HRD) 2007-2013, financed from the European Social Fund and by the Romanian Government under the contract number POSDRU/107/1.5/S/82839.
